# Why elderly in rural China didn’t wear masks during the COVID-19 pandemic? A qualitative narrative interview study

**DOI:** 10.1186/s12889-023-16653-0

**Published:** 2023-09-09

**Authors:** Yunlai Liu, Chunyan Huang

**Affiliations:** 1 School of Media, Anqing Normal University, Anqing, China; 2 The Department of Communication, University of Macau, Macau, China

**Keywords:** COVID-19, Masks, Rural elderly, Influencing factors, Qualitative

## Abstract

**Background:**

During the COVID-19 pandemic, ageism and stigmatization towards the elderly have been prominent issues. In addition, there have been debates on Chinese social media as to why elderly people in rural areas are not wearing masks. While some factors that affect the mask-wearing behaviour of the elderly have been analyzed, little attention has been given to the lived experiences and behavioral intentions of rural elderly people who choose not to wear masks, despite government mandates to do so.

**Method:**

In this research, 50 semi-structured interviews with 30 elderly individuals in three Chinese villages were carried out using the qualitative method of semi-structured interviews. Following verbatim recording and transcription of the conversations, the subject was analyzed using the Theory of Reasoned Action.

**Results:**

We identified four factors that influence the non-masking behaviour of rural elderly, including past experiences, cultural concepts, cognitive attitudes, and health and safety anxiety, and identified nine sub-themes based on the four overarching themes. Past knowledge, experience, and history have led rural elderly people to distrust the government's mandatory “mask mandate,” believing that they do not need to wear masks. Rural cultural concepts and habits make the elderly feel that masks not only fail to provide protection but also become obstacles, resulting in poor daily experiences. Cognitive attitudes and emotions determine the elderly's evaluation of masks, which in turn affects their use of masks. Finally, elderly individuals’chronic diseases directly affect the physical pain and life safety caused by their use of masks, which is a major objective factor for their non-masking.

**Conclusions:**

Although numerous studies have concluded that Chinese people wore masks out of collectivism and conformity during the pandemic, marginalized groups' opposition to wearing masks also contains distinctive, individualized elements and underlying causes. By exposing these elements and reasons, we can better comprehend the peculiar behavior of particular groups while fighting the pandemic. The needs of marginalized populations should be prioritized by public health policy makers to provide more equitable services.

**Supplementary Information:**

The online version contains supplementary material available at 10.1186/s12889-023-16653-0.

## Background

The rural elderly, as one of the most vulnerable populations, both in terms of coronavirus susceptibility and the mental health consequences of COVID-19 and related policy interventions such as social distancing, has received timely academic attention [1]. The incidence of COVID-19 infections and fatalities have increased in rural regions due to a number of variables, including poverty, unequal access to medical resources, and inadequate coverage of health information dissemination. According to surveys on COVID-19 infection, awareness, attitudes, and protection in rural Mexico [2], Bangladesh [3], India [4], and China [5], elderly people are more at risk of infection in rural regions than in urban areas. In China, there are more than 200 million people over the age of 60, and 75% of them reside in rural areas with limited access to healthcare and slow economic growth. They are vulnerable to infectious diseases and high-risk groups due to their weakened immune systems, and the majority of them are elderly patients who are severely ill as a result of the COVID-19 epidemic. Death rates for those 65 and older are 3.25 times higher than for those 55 to 64, and 26 times higher than for those under the age of 54 [6]. To better protect the elderly in rural areas from the virus, the Chinese government has taken a number of measures to contain the epidemic in rural areas, the most important of which is the implementation of a strict mandatory wearing of masks in public places, known as the mask mandate. In areas at high risk of the epidemic, those who do not wear masks are banned from public places and public transport systems. Refusal to wear a mask is officially considered an offence, with offenders being admonished or exposed through the media [7]. According to media reports, an older man in Shijiazhuang City, Hebei Province, who did not wear a mask outside during the pandemic was roughly tied to a roadside tree and verbally humiliated [8].

Therefore, during the epidemic, China’s Guangming Daily published a commentary by official scholar Zhou criticizing the rural elderly for not wearing masks, arguing that the “naked mouth” problem of the elderly encountered in rural epidemic prevention work is not only a manifestation of “lack of knowledge of virus transmission” but also “lack of civilized nutrition” and “lack of social morality [9]. On Chinese social media, young netizens attribute the reluctance of rural elderly people to wearing masks to stubbornness, intolerance, selfishness, backwardness and lack of public ethics, forming stereotypes and age discrimination against rural elderly people. They think “older people have gone bad” [10].

Why do older people in rural China choose not to wear masks during the “mask mandate”? Studies have provided different answers. Zhu et al. found that factors influencing mask wearing among rural elderly individuals included perceived vulnerability to health, perceived severity of health threats, behavioural efficiency to reduce health threats, and self-efficacy [11]. According to Meng’s findings, they do not wear masks because of their low education level, poor information communication and weak awareness of epidemic prevention [12, 13]. Research also suggests that factors influencing mask-wearing among rural elderly are also related to their past experiences. For example, a questionnaire survey of rural residents in Henan, Shanxi, and Shandong provinces in China found that the proportion of villagers wearing masks during the non-COVID-19 epidemic was only 14.89%, athough masks have been promoted as protection against infectious diseases since the plague in northeastern China in 1910 and the SARS pandemic in 2003 [14]. China has not developed a mask culture like South Korea and Japan. In Korea, it has long been a common practice for Koreans to wear masks, similar to wearing clothes [15]. In China, during the SARS pandemic in 2003, only a few people wore masks on the streets of Shenzhen, a city sandwiched between the two hardest-hit cities of Hong Kong and Guangzhou [16].

Although the survey data and findings explain the factors that influenced rural older people not to wear masks during COVID-19, there is a lack of research that investigates the logic behind epidemic prevention behaviour and examines its underlying causes among older people as subjects. There have been few studies that link these micro-logics to the personal habits, social and cultural backgrounds, physical attributes, and perceived attitudes towards masks of rural older people to examine the relationship between marginalised groups and epidemic prevention and control. Moreover, the left in Chinese academia regards the non-masked elderly as destroying collectivism and solidarity, and tries to push them onto the opposite side of the universal fight against the epidemic [17]. According to Cao, the absence of perspectives from the elderly in academic research has resulted in them becoming the “silent protagonists” during public health emergencies [18]. Exploring the cultural and social factors that influence mask wearing choices among rural elderly people requires an investigation into the factors and reasons that affect their choices. This will offer insights for future policy makers in developing public health policies tailored to this population.

This research examined the factors that prevent elderly individuals in rural China from wearing masks by means of qualitative semi-structured interviews and utilizing the Theory of Reasoned Action (TRA) to identify both direct and indirect influences. The Theory of Reasoned Action (TRA) was initially suggested by Fishbein and Ajzen during the 1970s to explain the relationship between attitudes and volitional behaviour [19]. According to the theory, a person's attitude serves as the immediate cause of their actions, while individual background factors, such as values, cultural environment, past experiences, and daily life, influence behaviour via norms and attitudes. In particular, personal experience has a great influence on individual behaviour, and people tend to decide on a certain behaviour based on their self-perception of previous attitudes. With the TRA, we can explain the choice of rural elderly people to wear masks and the underlying reasons behind it. Mask-wearing behaviour is an important public health measure to prevent the spread of the COVID-19 virus. In rural areas, the decision to wear a mask is influenced by various factors, including individual characteristics, subjective norms, life experience, physical condition, and emotional state. Therefore, it is necessary to identify the various factors that influence the decision of rural elderly people not to wear masks, rather than simply explain the impact of wearing masks on social cohesion and collective health. This study also provides a deeper understanding of the behavioural choices and decision-making processes of rural elderly during the epidemic. It also reminds us that when formulating and implementing public policies, we need to consider the needs and characteristics of different groups, and provide more support and attention to the most vulnerable groups.

## Methods

### Participants

In this study, a combination of targeted and random sampling was used to draw samples, and Xinyang City, Henan Province, was selected as a sample of our central traditional agricultural province, Baoji City, Shanxi Province, was selected as a sample of northwest inland agricultural area, and Beihai City, Guangxi Province, was selected as a sample of the southwest coastal agricultural area. Two representative villages were selected in the target area, one of which is the X village, home to two authors of this research, while the other is the Z village. Subsequently, a random sample was taken from a village, named Y village, which the authors were not familiar with, to supplement the problems and discoveries in the field investigation. This step aimed to improve the representativeness and universality of the research results. X village is situated in close proximity to Wuhan, the first city in China where the COVID-19 outbreak emerged, and was affected relatively early by the epidemic. On the other hand, Z village is located in a border area, which is crucial for epidemic prevention and control. The three villages share several common characteristics, such as their dependence on agriculture as their primary source of income, slow economic development, a noticeable ageing population with many elderly people left behind, and a relatively dense population. During the epidemic period, all three villages went through a period of “lockdown”. Despite these similarities, the villages are located in different geographic regions throughout China and display differences in population size, cultural background, lifestyle, infection status, and epidemic prevention and control policies. As of April 2023, according to unofficial statistics, the cumulative number of COVID-19 infections in Village X is 17, in Village Y is 105, and in Village Z is 124. During field investigations, we found that after China lifted all epidemic prevention and control measures on December 7, 2022, the number and rate of infections among rural villagers have been on the rise, but the number of people wearing masks has sharply declined, with elderly people almost “bare-lipped” when going out.

To ensure a diverse sample, two researchers (Liu and Huang) randomly selected 10 elderly individuals aged over 60 from three different villages for interviews, with the aim of cross-verification. Out of the 30 interviewees, 20 were male and 10 were female. The age of the interviewees ranged from 60 to 80 years old. Table 1 displays their demographic information and characteristics. The content and purpose of the interviews were verbally conveyed to the interviewees, and their consent to record the conversations was obtained. We conducted a total of 50 interviews, each with a duration between 30 and 70 min, with an average of 42 min. At the end of each interview, we thanked the interviewee for their contribution and offered daily necessities as a token.

**Table 1:** Characteristics of participants who participated in an interview (*N*=30)

		**Interviewee**	**Age**	**Gender**	**Chronic disease**
**Village**	X	X01	61	Male	No
		X02	65	Male	Hypertension
		X03	63	Female	No
		X04	67	Male	Cardiac disease
		X05	71	Female	Diabetic patients
		X06	65	Female	Respiratory disease
		X07	71	Male	Cardiac disease
		X08	72	Male	Gout
		X09	60	Female	No
		X10	62	Male	Hypertension
	Y	Y01	66	Male	Respiratory disease
		Y02	63	Male	No
		Y03	69	Female	Anemia
		Y04	68	Male	Cardiac disease
		Y05	73	Male	Diabetic patients
		Y06	71	Female	No
		Y07	72	Male	Gastrosia
		Y08	78	Female	Gout
		Y09	80	Male	Hypertension
		Y10	75	Female	No
	Z	Z01	60	Male	No
		Z02	62	Female	No
		Z03	74	Male	Coronary disease
		Z04	71	Female	Anemia
		Z05	83	Female	Rheumatism
		Z06	60	Male	No
		Z07	77	Male	Respiratory disease
		Z08	62	Male	No
		Z09	68	Male	Hypertension
		Z10	65	Male	Hyperlipidemia

### Data collection

The interviews were conducted in a semi-structured format, face-to-face and one-to-one, with the researchers guided by the guideline questions (see Additional file 1). Liu, the researcher, was responsible for following up and interviewing 20 elderly people in villages X and Y, while Huang interviewed 10 elderly people in Village Z. The interviews were conducted in two periods. In the first phase, from January 2022 to May 2022, Chinese provinces and cities are in a state of continuous multi-point outbreak, and the three villages mentioned above are intermittently enforcing the “mask mandate” along with the peaks and valleys of the outbreak. The purpose of this phase of the interviews was to focus on the rural elderly’s perceptions, attitudes, and daily wearing of the “mask mandate”, and to explore the factors that influence their wearing of masks and how these factors relate to their habits and cultural background. The second phase is from February 2023 to April 2023. On January 8, 2023, China fully lifted the preventive and control measures against the new coronavirus infection, social mobility and daily production return to normal order, and the requirement to wear masks in public places become increasingly relaxed. The purpose of this phase of interviews was to interview 30 rural seniors to re-examine their perceptions of the epidemic, masks and prevention and control policies, and to learn more about whether the three-year-long mask mandate had an impact on them. After the interviews were completed, all audio recordings of the interviews were transcribed verbatim into written transcripts. For interviewee privacy, identifiable names and related information were anonymized, and all interviewee names were alphabetically numbered.

### Analysis

Following Fishbein and Ajzen's interpretation [20], Liu and Huang, the researchers, utilized NVivo12 to annotate and categorize the transcribed materials, with the guidance of the TRA theoretical framework. The researchers extracted and summarised text themes, enciphered acquired themes, assigned unique codes to each theme, established an encoding system, and conducted statistical analysis and interpretation of the enciphered outcomes. This was an iterative process, and the researchers frequently discussed newly discovered codes that were not captured by the original encoding framework. Liu and Huang reviewed and discussed the encoding procedure (see Additional file 2), progressively concluding on the themes related to the research.

## Results

According to the TRA, four main themes were identified: (1) past experiences, (2) cultural concepts, (3) cognitive attitudes, and (4) health and safety anxieties. Based on these four overarching themes, we have defined nine sub-themes which are detailed in Table 2.

**Table 2:** Themes and sub-themes identified in the interviews

**Theme 1: The experience and impression towards masks in the past life**
(i) Distrust and lack of understanding of the mask mandate (ii) Connecting face masks with health care workers
**Theme 2: Cultural concepts and living habits of rural society**
(i) Death and life are determined by fate, riches and rank decreed by Heaven (ii) Living habits in rural areas (ii) Respect the elderly and take advantage of one’s seniority
**Theme 3: Personal cognition and attitudes of older adults**
(i) Fluke mind (ii) Dissatisfaction with the prevention policies
**Theme 4: Chronic diseases and emotional anxiety of older adults**
(i) Chronic diseases and physical health safety (ii) Anxiety of lockdown

### Theme 1: previous impressions of face masks and experience of wearing masks

Leone argues that medical masks are familiar and not entirely new in the East, especially in Japan, China, and Korea [21]. However, in the three villages investigated in this study, there was very little exposure to masks before the COVID-19 outbreak. One reason for this is that farmers involved in agricultural production rarely require the usage of masks in their productive activities and daily lives. The only thing that impressed them about masks as medical supplies for defense against infectious diseases was the SARS outbreak in Guangzhou in 2003. As a result, in terms of mask awareness and familiarity, the residents in these three villages are still caught in the past. Most villagers are unfamiliar with the requirement to wear masks.

### Distrust and lack of understanding of the mask mandate

When COVID-19 broke out in the seafood market in Wuhan in early 2020, villagers in Y village, thousands of miles away, were preparing for the Spring Festival. At the beginning of the pandemic, the village elders did not know about the virus and only learned about it through TV news and interpersonal communication. Because of low information contact and distance, most elderly people think the virus does not threaten their normal lives. They did not accept the measures taken by the government and doubted about the mask mandate.

“I am reluctant to wear a mask. No one in the village has worn masks for the past few decades, so who can prove that masks are effective in preventing viral infections?” [Y02]

Y05 recalled that on the night of the first day of the Spring Festival in 2020, the village imposed a lockdown. As a governmental action, village “lockdown” represented the implementation of national-level measures to control the pandemic in rural areas. This critical and organized action enhanced villagers’ perception of the risk of the pandemic and sent the message that the severity of the pandemic was escalating. Although rural elders were aware that the epidemic was spreading rapidly, they were still making judgments based on their personal previous experiences.

“In the elderly’s experiential perspective, COVID-19 appears to be no different from previous flus, and therefore there is no need to wear masks.” [Y05]

Researcher Liu found in the Y Village interviews that older individuals’ past behaviours and experiences have a significant impact on the actions they would take. The past experience of not wearing masks in SARS influenced their attitude towards the implementation of the mask mandate during the COVID-19 period. This result confirms Yoo and Lee’s research, which suggests that people’s past behaviour has a significant influence on their present and future behaviour [22].

### Connecting face masks with health care workers

One of the most frequently reported issues by participants was that masks were directly associated with doctors and nurses in their past experiences and impressions. Before the pandemic outbreak, the farmers’ empirical perception was that medical protective masks were worn by nurses, doctors and other healthcare workers. In their experience of SARS, the mainstream media at the time called for “saving masks for the doctors and nurses who needed them most”. Masks are usually not required for anyone other than medical staff, those in close contact with infectious illnesses and those visiting hospital patients.

“Farmers have been working hard in the fields for generations. They are not doctors or nurses, do they still need to wear masks? Have you ever seen farmers wearing masks while tilling the land?” [X03]

Participants’ statements suggest that the mask-wearing behaviour of the elderly during the COVID-19 pandemic was influenced by the individual's past experiences. The discursive narratives of the elderly based on their personal subjective experiences and the symbolic meaning given to the use of masks are difficult to be fully understand by young social media users or policy makers. Past experiences have prompted farmers to symbolize masks and associate them with specific occupations and specific environments. They chose not to wear masks in the pandemic period because they had done so before in similar situations.

### Theme 2 Cultural concept and living habits in rural areas

TRA has been effectively applied in different cultures, but these studies confine culture to a binary framework including certainty and uncertainty, masculinity and femininity, individualism and collectivism. All these studies emphasized that people’s cultural values can be used to explain behavioural differences [23]. In Chinese rural society, individualism and collectivism can also influence farmers’ behaviour in wearing masks. In addition to these two factors, the health and philosophy of survival of elderly people should be considered. When these cultural concepts and principles of life meet the mask mandate of the national epidemic prevention policy during the epidemic control period, the elderly will make their own choices according to their own expectations, despite the policy and moral pressures that often come with them.

### Life and death are destined by fate, rank and riches decreed by Heaven.

“Life and death are destined by fate, rank and riches decreed by Heaven” is from the Chinese Confucian classic “Analects of Confucius—Yan Yuan”. It refers to the fact that everything that happens to people, such as life and death, is determined by destiny, and we cannot fully control our own life and death and wealth, but can only do our best. This is a philosophical view of objective idealism and a cultural perception that exists in Chinese rural society. Rural elderly people have worked hard all their lives, and they look at success or failure very lightly. In their view, the life span of people living in the world, diseases, are beyond their control, and people cannot be changed, especially if they face death, no one can stop it.

“As the saying suggests, life and death are predetermined, and prosperity and abundance may be considered as blessings from above. The aging, sickness, and death of people are predetermined. In situations beyond their control or awareness, people should indulge themselves. As the saying goes, man proposes, God disposes.” [Y10]

According to the literature, wearing a mask during the outbreak in China was seen as a collectivist and morally commendable decision—associating it with demonstrating “small love” for family and “big love” for one’s country [24]. Particularly in mainstream media coverage, there has been an attempt to construct the mask as a unifying force for all citizens and businesses, with ‘wearing a mask’ as a metaphor for solidarity and social responsibility [25]. However, in China’s people’s war against COVID-19, the highly centralised system and the top-down approach to policy design and implementation were not always consistent when finally implemented in the countryside, and the reflection of grassroots society was sometimes fragmented [26]. One of the factors in the different forms of disobedience by urban and rural villagers is the conflict between the overall strict policy of epidemic prevention and the differentiated cultural perceptions of individual villages. When the coercive policy, the collectivist moral pressure, requires the rural elderly living in the grassroots to wear masks, it often ignores the individual diversity of life experiences and cultural concepts. In this war against COVID-19, rural elders should not be seen as passive victims in need of protection, but as active individuals.

### Living habits in rural areas

During the interviews, we discovered that rural elders’ habits and behaviors are shaped by particular historical circumstances and living environments. Y village is located in western China, where economic development has historically been backwards, with little drought, little rain, a lot of sand, and insufficient water resources. People’s living conditions are relatively poor, and the elderly people rarely have the opportunity to take a bath. It is difficult to execute the protective measures of “frequent hand washing” and “frequent ventilation” in this living environment. In their everyday activities, local villagers have a habit of gathering to chat. In the rural “acquaintance society,” villagers frequently visit each other, talk, and play mahjong. In such a situation, implementing the epidemic prevention policy requirements of “washing hands regularly,” “frequent ventilation,” “no gathering,” and “wearing masks” in Y village is challenging.

“In our rural areas, during windy weather, sand and dust swirl everywhere, finding their way into our ears, eyes, and mouths. In such conditions, it is not uncommon for people to go without masks. However, due to the COVID-19 outbreak, the wearing of masks has become mandatory, which I find uncomfortable and cumbersome. While engaging in physical labor, the restricted airflow and heat trapping properties of the mask make it difficult to breathe and beads of sweat collect on my face.” [Y07]

These quotations form the interviewers remind us that the relevant literature, amidst the heated debate on whether to wear a mask or not [27], has paid little attention to the conflicts that individuals within China, especially the rural elderly, provoke with their new habits when their old ones are reconfigured. For example, requiring older people to wear masks to do farm work is completely inconsistent with their long-standing work habits.

### Respect the elderly and take advantage of one’s seniority

Respect for the elderly has been handed down in Chinese culture for thousands of years. In Chinese culture, the elderly have made contributions to the country, society and family and are worthy of respect. Even before the pandemic, Chinese society had developed an atmosphere that gave the elderly an honoured status and priority in public life. However, during COVID-19, there were unfriendly attitudes and behaviours of the younger generation towards the elderly. Criticism is widespread on social media, accusing the rural elderly of being “old age”, “ignorant”, “selfish”, “unpatriotic”, “indifferent to epidemics” and “troublemakers”. There have been numerous media reports of rural elderly people being beaten up for not wearing masks, being verbally abused and chased away by supermarket security guards, and even dying of rage during fights [28]. Although these are extreme cases of conflicts between the younger generation and the rural elderly during COVID-19, they reflect the cracks in the culture of respect for elderly individuals, which has been passed down for thousands of years under special circumstances.

“Now I don’t like to go out on the street. Young people can’t say that we are creating chaos and criticize us for being old because the elderly don’t wear masks. I think this is not fair, lost the minimum respect and care for the elderly, do not forget, this is our traditional virtues.” [Z04]

The original meaning of leaning on the old man is that the old man relies on his age, relies on his experience and merit to handle things subjectively, and belittles or ignores others. Some of the older participants felt that it was unreasonable for rural elders to be criticised by young people for relying on elders when they refused to wear masks during the epidemic. The reason is that not wearing masks is not because they are rude and unreasonable, but because they believe it is the ‘right’ choice in the traditional culture of respect for elders. For a long time in the past, the tradition of respecting elders ensured that rural society and individual families had an orderly and harmonious development of elders and children. In the opinion of the elderly, despite being in the midst of rural epidemic prevention and control, the tradition of respecting the elderly cannot be lost, and they should be given greater tolerance in the issue of wearing masks.

### Theme 3 Personal cognition and attitudes of older adults

Individual subjective cognition plays an essential role in TRA, explaining the role that individuals’ perceptions of external things play in people performing a certain behaviour. Although the positive effect of individual subjective perceptions on behaviour has been documented, the role of rural elderly people’s perceptions on mask behaviour during the epidemic was not necessarily always positive [29]. For Chinese people, the face, collectivism, and other moral norms of rural acquaintance society can have a normative effect on the behaviour of elderly people wearing masks, but this does not mean that collective goals should be given more importance than individual perceptions and feelings in a collectivist society. Personal cognition also affects behaviour indirectly by influencing attitudes. According to Ajzen and Fishbein, attitudes represent an individual’s evaluation of some aspect of the world, such as another person, a physical object, a behaviour, or a policy [30]. Some people have positive attitudes toward wearing masks, while others have negative attitudes, and people’s attitudes continue to influence their behaviour.

### Fluke mind

During the interviews, some of the elderly were aware enough of the seriousness of the epidemic that they needed to wear masks when they went out; however, others were equally aware of the dangers of the pandemic, but still gathered to chat or entertainment without masks. During the interviews in village Z, no COVID-19 infected patients were found in the village, but infected patients were found in other villages in the same city at that time. As a result, some Z villagers believed that the virus would not come to their village and habitually relaxed their vigilance. The scene of village elders gathering to play mahjong without masks reflects the villagers' attitude of not actively protecting themselves. When asked if an elderly man wore a mask, he said:

“The people were still wearing masks a few days before the village locked down. However, the number of persons using masks drastically decreased after a few days. Why bother wearing a mask in such a small village where everyone knows each other so well? I don’t think I’m susceptible to infection at my senior age.” [Z03]

The fluke minds of the rural elderly have made them “confident” in the face of COVID-19. Under the influence of this fluke, they voluntarily give up self-protection. Based on this, the elderly develop an “optimism bias” [31]. They tend to underestimate their own vulnerability to risk and overestimate the sensitivity of others to risk. This mentality is influenced by the long-established closed and stable characteristics of rural society and the interpersonal relationships in the “acquaintance society”, which have existed for many years in rural society, and it is difficult for the elderly living in rural areas to change their perceptions in a short period of time.

### Dissatisfaction with the prevention policies

Chinese society is characterised by remarkable civic obedience and a high degree of social discipline, according to some studies [32]. Citizens are required to exercise restraint when individual interests conflict with collective interests [33]. Both views see all Chinese as ‘the silent lamb’. However, there are hidden grievances and resistances in various social events and policy implementation. In April 2022, the spread of the pandemic in China reaches a new peak. In some areas, rural governments are forcing villagers to stay indoors to reduce their mobility, requiring them to stay at home and plant at different times. Farmers must apply for a ‘spring farming permit’ to enter their fields and work. There are 20 of these permits issued per day per village, and the issuer is limited to the village head. In Anshan City, Liaoning Province, farmers are even required to wear protective clothing when ploughing and sowing in the fields. Similar outbreak prevention and control policies, although not generalised in rural areas, provide a glimpse of the disruptions that some of them have also brought to the productive order of farmers lives. The lockdown policy and mask mandate easily provoked the discontent of elderly individuals.

*“*We are farmers, is it reasonable that we still need permission to work in our own fields? If we are not allowed to grow crops, what will we eat?” [X07]

Although official publicity emphasises that the elderly are physically weak and have little resistance, it is all the more important for them to wear masks for self-protection, and they may face death in case of infection. However, for the elderly, the ongoing lockdown policy has had a negative impact on their lives and financial incomes. Especially for low-income elderly groups who depend on agricultural production, the epidemic prevention and control policy has plunged them into poverty, leading to dissatisfaction with the policy. The most direct manifestation of this is the reluctance to wear a mask, or the formality of wearing a mask that covers the mouth but not the nose.

### Theme 4 Chronic diseases and emotional anxiety of older adults

Hanna et al. investigated the physical-related problems caused by community residents wearing masks during the COVID-19 pandemic. For example, wearing masks in winter caused their glasses to fog up, which affected their vision and safety; wearing masks in summer caused problems with their thermoregulation; and difficulty breathing while wearing masks severely limited their outdoor exercise [34]. The results of this study suggest that the negative issues related to people's health and safety caused by wearing masks are also important factors that influence people’s decision to wear masks. This study further reveals that having a chronic disease in rural areas also affects the elderly people’s willingness to wear masks. For the elderly in rural areas who are physically weak and lack health maintenance, wearing a mask may induce a chronic disease attack at any time, thus endangering health and life safety.

### Chronic diseases and physical health

“My wife and I have both had high blood pressure for over 10 years. We must take blood pressure medication every day, as missing a day could put us at risk. With these basic diseases, we usually rarely wear masks. When we wear masks during travel, we get panic attacks, dizziness and breathlessness.” [X05]

Yu pointed out that five chronic diseases have plagued farmers over the last decade in rural China. Hypertension, diabetes, ischemic heart disease, cerebrovascular and lung diseases are the main chronic diseases. The causes of these physical diseases are related to their experiences of starvation during adolescence and the physical damage caused by long-term and continuous exertion in adulthood [35]. In a Chinese media report, a village in Hunan Province had 504 people over 60 years of age in early January 2023. More than 310 were infected, and four of them died during the infection [36]. Combining Yu’s research and media reports would suggest that rural elderly individuals, as a susceptible and high-risk group for COVID-19, should have protected themselves by wearing masks. However, chronic disease affects their behavior of wearing masks. This contradiction often tends to undermine their mood and cause psychological anxiety.

“I suffer from bronchitis and find it difficult to wear an N95 mask. After walking a certain distance, I experience difficulty breathing. I am concerned that wearing a mask may exacerbate my condition.” [X08]

In addition, respondents also reported other adverse physical reactions caused by wearing masks. For example, an elderly man with diabetes felt that wearing a mask was too stuffy, thus causing a sudden drop in his diabetic sugar level; an elderly man with heart disease and coronary heart disease felt that wearing a mask for a long period of time made it difficult for him to breathe and tended to cause a faster heartbeat and heart pressure to prevent him from breathing properly. As a result, some seniors wear masks that can trigger varying degrees of physical health and safety issues, ranging from minor inconvenience to causing significant problems in daily life and even preventing seniors from performing daily activities. In such cases, seniors may choose not to use masks.

### Anxiety of lockdown

Several studies have shown that home isolation during COVID-19 caused social isolation and psychological sequelae in elderly individuals, which led to a variety of adverse physical health and psychological disorders, including depression [37–40]. However, these studies ignore the relationship between the anxiety of lockdown and the mask-wearing behaviour of the elderly during the outbreak. We found in our interviews that wearing masks affected the emotions of the rural elderly because they still gathered at the call for lockdown, and wearing masks affected their communication with each other.

“The requirement to wear masks and stay-at-home orders have caused the elderly to experience a loss of collective entertainment and social interaction. Less time spent outside has led to disruptions in sleep patterns. They feel lackluster and are unhappy, restless, and anxious.” (Y08)

Wearing masks affects seniors’ daily activities, including significantly reducing the frequency of travelling, going shopping and entertaining. For the elderly in rural areas, wearing a mask is difficult. To avoid wearing masks, they had to go outside less often.

## Discussion

Our research employs qualitative analysis to address a highly disputed issue: why did elderly people in rural areas not wear masks during the COVID-19 outbreak in China? On the one hand, the spread of this question created ageism and stigmatization of the elderly in social media, and on the other hand, it showed that the discourse and behavioural logic of epidemic prevention among the rural elderly is missing and invisible. This imbalance objectively causes the elderly, who are already vulnerable themselves, to become victims again. This study found that the behaviour of elderly people not wearing masks is closely related to their past historical experiences, cultural values and habits in a particular environment, personal cognitive attitudes, and physical health and safety. The results of this study reveal that individuals do not always exhibit a high degree of compliance with China’s national policy. In the face of highly centralized power and strict lockdown policy and masking mandate, they were still able to make their own behavioural choices based on their own cultural perceptions, survival logic, cognitive attitudes, and empirical judgments. Previous studies have emphasized the importance of wearing a mask to stop the transmission of infectious disease through droplet transmission [41]. We also need to consider whether individuals exhibit unique COVID-19 prevention behaviours within the context of the symbolic metaphor of collectivism and solidarity represented by mask-wearing. Individual uniqueness is not only related to individualism, but it also embodies the judgments that individuals make based on their own realities, and this judgment further influences their behavioural intentions.

In our sample, 30 elderly people in three villages gave their own reasons and motivations for not wanting to wear a mask. They did not want to fight the state’s epidemic prevention policy or undermine society, as the younger generation criticises. They chose not to wear masks because they wanted to maintain their lifestyles, social interactions and health. One of the aims of this study is to present the uniqueness of this group in order to appeal for understanding and tolerance of the behaviour of the elderly in not wearing masks in the conflict of “wearing masks or not", and to change the age discrimination of “the elderly are becoming bad".

Another contribution of this study is that the analysis of factors influencing the behaviour of rural elderly people who do not wear masks can provide lessons for the implementation of other epidemic prevention policies in the future. Some studies view mask wearers as good citizens who are responsible and follow the rules, have a better chance of survival than non-compliant wearers, and see obedience to authority as a life-saving and moral right [42]. In contrast to this discourse, we recognise the importance of wearing masks as a protective measure and a moral responsibility. However, we are also wary of state and media narratives that associate the wearing of masks with authoritarianism, creating a divide between those who wear masks and those who do not conform to policies and public expectations. During the pandemic, Agamben was critical of the government’s overreaction, particularly the imposition of a state of emergency in peacetime. He repeatedly pointed out that the state of emergency is a mechanism by which democracies become totalitarian societies, and that governments use it as a tool to manage so-called “worst-case scenarios” by creating a “health terror” [43]. During periods of uncertainty, the desire to curb the spread of the pandemic and a sense of civic responsibility are among the key motivators for individuals to wear masks. However, it is equally important to pay attention to the perspectives of special groups, such as the rural elderly. Some elderly individuals may have made a different choice under such a “mask mandate”. Understanding their reasons can help us better cater to the needs and concerns of all members of our society. Policymakers need to take into account the dichotomous characteristics presented by differentiated groups in epidemic preparedness and public health measures, for example, some groups feel safer wearing masks, while others report that wearing masks is anxiety-inducing and health-threatening.

Despite the existing literature indicating that the benefits of wearing masks may outweigh any other negative consequences, the negative consequences of wearing masks among rural elderly people, such as negative emotions [44], induction of chronic diseases, habitual discomfort and emotional anxiety, should not be ignored. For these elderly respondents, the many problems caused by wearing masks are not trivial; rather, their effects can threaten the daily lives and physical health of the elderly. Ignoring the threatening nature of these negative consequences may lead to the marginalisation of these groups by society and epidemic prevention policies. This outcome would result in social exclusion during efforts to strengthen epidemic prevention policies during a pandemic. Therefore, as a grassroots management organisation implementing communicable disease prevention policies, it is crucial to identify the needs of special groups before designing and formulating policies.

While our qualitative approach allowed us to identify factors that influence rural elders’ decisions to wear masks, our study has some limitations. First, we acknowledge that our sample was limited to a specific geographic location and may not be representative of rural elders in other regions. Second, the impact of the epidemic infection on our participants’ attitudes towards wearing masks may differ from those of more urban middle-aged and older populations. Furthermore, future studies could include larger random samples with more diverse age groups to increase the generalizability of findings.

## Conclusion

**Fig. 1:**
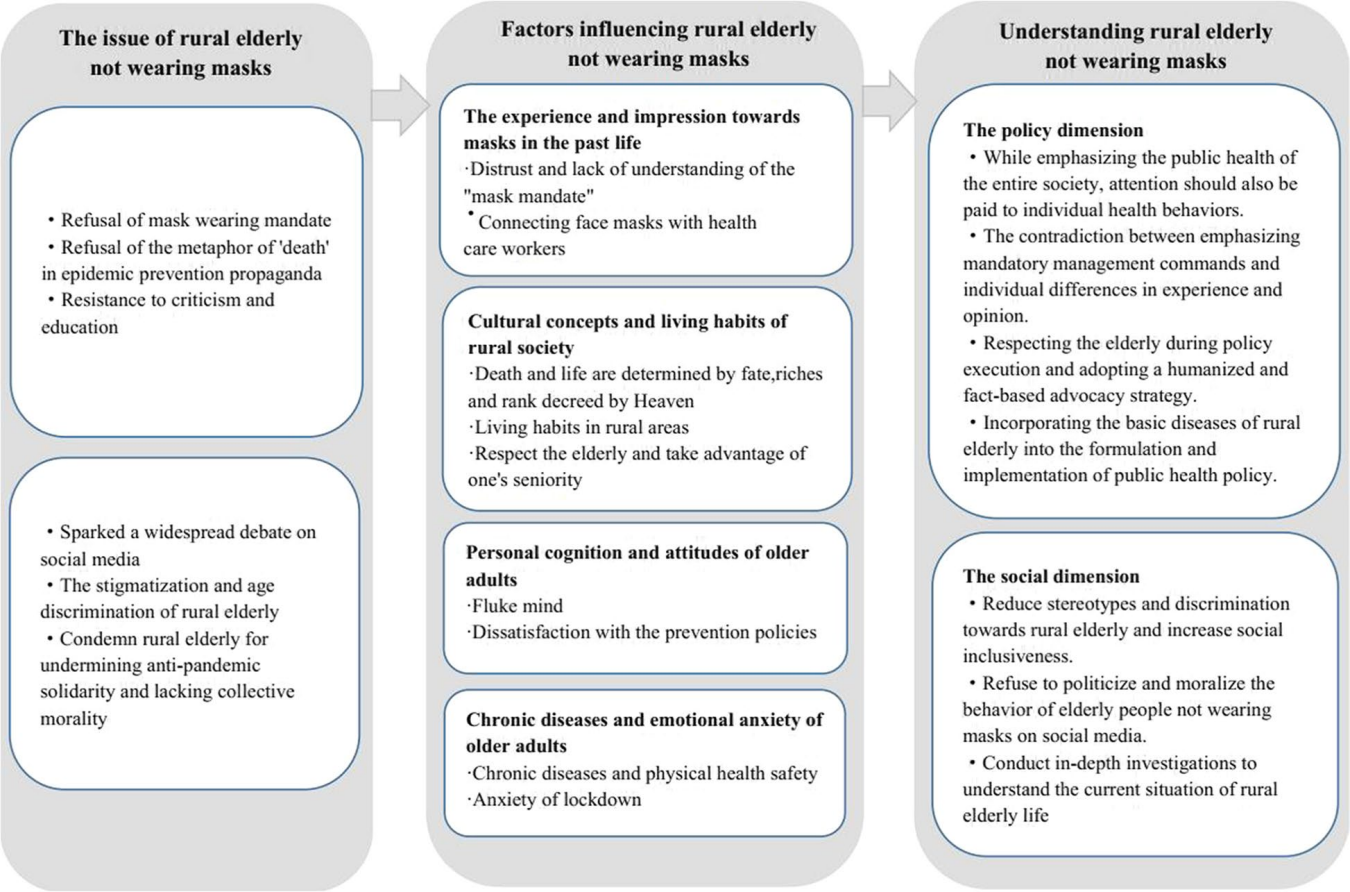
Why some rural elderly choose not wearing masks during the COVID-19 pandemic

Figure 1 below summarizes the key insights of the study and provides evidence that some rural elderly in China refuse to wear masks, which corresponds to the popular topic on Chinese social media of “Why don’t rural elderly wear masks?”. The study’s findings reveal the reasons why elderly rural residents are resistant to wearing masks during the mask mandate and outline some potential risks associated with this behaviour, particularly for elderly individuals with chronic illnesses, as wearing a mask may lead to new health issues for them. This study helps to gain a deeper understanding of topics related to TRA and enriches the application of TRA. As depicted in Fig. 1, our findings highlight the importance of group-specific cultural perceptions and cognitive attitudes in comprehending public health interventions and serve as a reminder to policymakers to choose mask mandates in the epidemic prevention process in a fair and reasonable manner to ensure that public health policies take into account the needs of marginalized groups. During the COVID-19 pandemic, wearing masks is a beneficial health behaviour for the entire society, but it is also a personal health behaviour. Therefore, while paying attention to public health policies, factors influencing mask usage should be studied from an individual perspective, with particular emphasis on individual subjective norms, living environment, and past experiences. In the formulation of public health policies, measures with humanistic care should be adopted, instead of a one-size-fits-all approach. At the societal level, efforts should be made to reduce stereotypes about rural elderly people and to provide them with emotional and psychological support.

### Supplementary Information

**Additional file 1. **Topic guide for semi-structured qualitative interviews.

**Additional file 2. **Sample coding process.

## Data Availability

The datasets used and analyzed during the current study are available from the corresponding author on reasonable request.
