# Supplementary material for: Why elderly in rural China didn’t wear masks during the COVID-19 pandemic? A qualitative narrative interview study
Source: BMC Public Health. 2023 Sep 9;23:1757. doi: 10.1186/s12889-023-16653-0 (PMC10492314; doi:10.1186/s12889-023-16653-0)
Supplement: Supplementary file 1 — Additional file 1. Topic guide for semi-structured qualitative interviews. [file 12889_2023_16653_MOESM1_ESM.docx]

**Additional File 1**

Topic guide for semi-structured qualitative interviews

**Introduction**

Hello! I'm very glad to see you.

Thank you for taking the precious time to accept our interview. We are Yunlai Liu and Chunyan Huang from Shenzhen University. We would like to ask you to share some of your opinion about wearing masks during the epidemic.

There are no wrong answers but rather different points of view. Please feel free to share your point of view, even if it differs from what others have said. Keep in mind that we're just as interested in negative comments as positive comments, and at times the negative comments are the most helpful.

We're recording the session because we don't want to miss any of your comments. We will use your first name during the interview, but they will not appear in our reports. You may be assured of complete confidentiality.

**Question Guide**

**General**

1 Can you tell me if you have heard or learned about Covid-19? How do you handle it in your daily life?

2 Can you tell me if there are any Covid-19 infected persons in your village? Are all the people in the village wearing masks?

**Past experiences**

1 You can tell me if you have ever experienced a major epidemic before?

2 Can you tell me if you have ever worn a mask before, and if so, was it a regular mask or a medical mask?

3 Can you tell me whether you wore a mask before, and if it was a regular mask or a medical

one?

4 After lifting the "mask mandate" ,will you still wear a mask?

**Cultural concepts and living habits**

1 What does a mask mean to you?

2 Can you tell me your opinion on the Covid-19 pandemic?

3 Can you tell me about your experience wearing masks during agricultural production activities?

4 Can you tell me how various pandemic control measures have affected your life and work?

5 Can you tell me how you view young people's persuasion and criticism towards older people who do not wear face masks?

**Individual cognition and attitude**

1 Can you tell me whether you are willing to wear a mask and why?

2 Covid-19 epidemic, do you think wearing a mask has a preventive effect?

3 The Covid-19 pandemic has led to the death of some infected elderly people, are you afraid?

4 Layer-by-layer intensifying epidemic prevention policies will affect your decision on whether to wear a mask?

**Chronic diseases**

1 Can you tell me if you suffer from a chronic disease?

2 In daily life, wearing a mask will bring what uncomfortable sensations to your body?

3 Will home quarantine and mandatory mask-wearing bring you loneliness or anxiety?
