# Supplementary material for: Why elderly in rural China didn’t wear masks during the COVID-19 pandemic? A qualitative narrative interview study
Source: BMC Public Health. 2023 Sep 9;23:1757. doi: 10.1186/s12889-023-16653-0 (PMC10492314; doi:10.1186/s12889-023-16653-0)
Supplement: Supplementary file 2 — Additional file 2. Sample coding process. [file 12889_2023_16653_MOESM2_ESM.docx]

**Additional file 2**. Sample coding process

| **Meaning Unit** | **Codes** | **Subthemes** | **Themes** |
| --- | --- | --- | --- |
| *"If one mask can block the virus, why do we still need hospitals?"* | Trust | Do not trust and do not understand the "mask mandates." | *Past Experiences* |
| *“In my impression, only doctors wear masks.”* | Profession | Connect masks with medical professions |  |
| *“Every person has their own destiny, and they cannot avoid it even if they try.”* | Fatalism | Man proposes but god disposes | *Culture Concept* |
| *“I before eating not wash hands habit，unclean, eating disease！”* | Cultural habits | Rural social customs and habits |  |
| *"I don't wear a mask, what's wrong? Why can't young people show respect to the elderly?"* | Respect | Respect the elderly and take advantage of one's seniority |  |
| *“COVID-19 far away from us, I just don't believe can be infected.”* | Fluke | Fluke mind | *Personal Cognition and Attitude* |
| *"Farmers have to obtain a permit to cultivate their land, isn't this a joke?"* | Dissatisfaction | Dissatisfaction with some epidemic prevention policies |  |
| *"I suffer from hypertension and cannot wear a mask for a long time, otherwise I cannot breathe."* | Diseases | Chronic diseases and physical health safety | *Health and Safety Anxiety* |
| *“Originally, there was nothing wrong with my body, I was just worried that wearing a mask would cause me problems by suffocating me.”* | Anxiety | Home quarantine's loneliness and anxiety |  |
